# Unusual Cause of Right Upper Quadrant Pain: Hepatic Amoebic Abscess

**DOI:** 10.1590/0037-8682-0519-2023

**Published:** 2024-02-12

**Authors:** Elif Gündoğdu

**Affiliations:** 1 Eskişehir Osmangazi University, Faculty of Medicine, Department of Radiology, Eskişehir, Turkey.

A 66-year-old female, with no significant history, presented with a two-week history of right upper quadrant pain and chills. Her temperature was 36.8°C, and other vital signs were within normal limits. Physical examination revealed tenderness to palpation in the right upper quadrant. 

Laboratory tests showed leukocytosis (12.6×10^3^ uL), elevated C-reactive protein (45 mg/L), erythrocyte sedimentation rate (38 mm/h), procalcitonin (0.06 ng/mL). Liver enzymes were slightly elevated (AST: 35 U/L, ALT: 48 U/L, and ALP: 162 U/L). 

**FIGURE 1:**
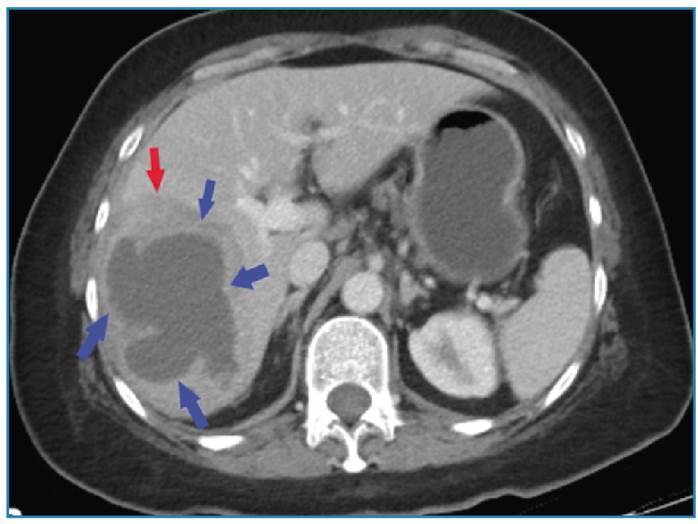
Computed tomography showing a thick-walled hypodense cystic lesion (blue arrows) with surrounding edema (red arrow).

**FIGURE 2:**
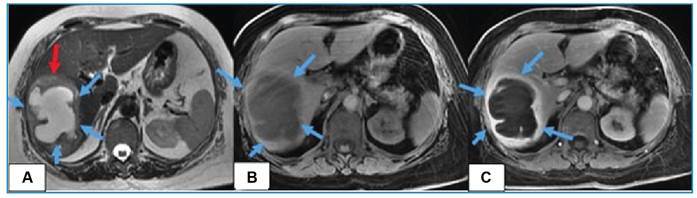
Magnetic resonance imaging scan showing a **A)** hyperintense lesion on T2-weighted imaging (blue arrows) with perilesional edema (red arrow), a **B)** hypointense lesion on T1-weighted imaging (blue arrows), with an **C)** enhancing peripheral rim-like thick capsule (blue arrows).

**FIGURE 3:**
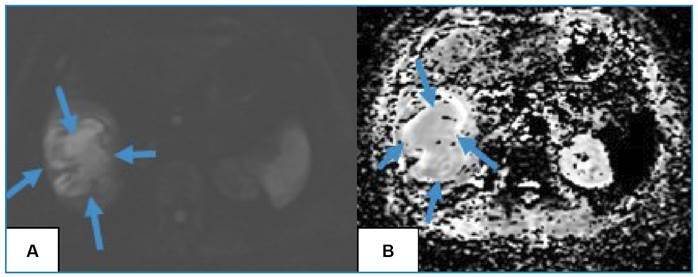
**A)** Diffusion-weighted images and **B)** apparent diffusion coefficient map shows restricted diffusion within the lesion (blue arrows).

A computed tomography scan revealed a thick-walled cystic lesion with surrounding edema in the right lobe of the liver (Figure 1). Magnetic resonance imaging was performed to confirm the diagnosis. It showed the abscess with its characteristic thick enhancing wall and diffusion-restricting content (Figure 2, 3). 

Based on the clinical presentation, imaging findings, and elevated inflammatory markers, a diagnosis of hepatic amoebic abscess was made. She was treated with metronidazole (500 mg) thrice daily for 10 days. Her symptoms significantly improved after initiation of treatment. Repeat imaging six months later showed a complete resolution of the abscess. While *Entamoeba histolytica* is a well-known pathogen^1^, causing potentially life-threatening hepatic amoebic abscess, its presentation can be atypical, as seen in this case. The extraintestinal disease is uncommon, and the liver (3-9%) is the most commonly affected organ^1^ ^,^ ^2^. Amoebic liver abscesses are more likely to be solitary than multiple lesions, and are more commonly found in the right lobe than in the left^3^. Drainage is not recommended because of the risk of rupture; medical treatment is preferred.
